# Pembrolizumab-induced focal segmental glomerulosclerosis: A case report

**DOI:** 10.1097/MD.0000000000027546

**Published:** 2021-10-29

**Authors:** Da Woon Kim, Hakeong Jeon, Sungmi Kim, Wanhee Lee, Hyo Jin Kim, Harin Rhee, Sang Heon Song, Eun Young Seong

**Affiliations:** a Department of Internal Medicine, Pusan National University Hospital, Busan, Korea; b Division of Nephrology, Department of Internal Medicine, Biomedical Research Institute, Pusan National University Hospital, Busan, South Korea.

**Keywords:** focal segmental glomerulosclerosis, immune checkpoint inhibitors, immune-related adverse events, pembrolizumab

## Abstract

**Rationale:**

Focal segmental glomerulosclerosis (FSGS) is the most common primary glomerular disorder that leads to end-stage kidney disease. Pembrolizumab, an immune checkpoint inhibitor, is an anti-programmed death 1 (PD-1) immunoglobulin G4 antibody approved for the treatment of advanced melanoma and can cause various renal immune-related adverse events (AEs), including acute kidney injury. Several cases of anti PD-1 therapy-induced glomerulonephritis have been reported so far, but FSGS has seldom been reported.

**Patient concerns:**

46-year old woman presented to our hospital with generalized edema.

**Diagnoses:**

Laboratory examination revealed features of nephrotic syndrome, and kidney biopsy confirmed FSGS. After other etiological factors of secondary FSGS were ruled out, she was diagnosed with FSGS caused by pembrolizumab.

**Interventions:**

She did not resume treatment with pembrolizumab and was treated with irbesartan and furosemide according to the American Society of Clinical Oncology Practice guidelines.

**Outcomes:**

After 2 months, the features of nephrotic syndrome resolved.

**Lessons:**

This case provides valuable insight into the etiology of FSGS that can occur as a renal immune-related AE of PD-1 inhibitor therapy. Therefore, patients should undergo evaluation for renal function and urinalysis at baseline and after treatment. If patients treated with PD-1 inhibitors present with renal injury and/or unexplained proteinuria >1 g/day, we would recommend a kidney biopsy to determine the underlying cause and establish an appropriate therapeutic plan.

## 1 Introduction

Focal segmental glomerulosclerosis (FSGS) is the most common primary glomerular disorder that leads to end-stage kidney disease.^[1]^ FSGS is a “podocytopathy” characterized by podocyte injury induced by various causes that leads to podocyte foot process effacement and nephrotic proteinuria.^[2]^ The etiology of FSGS is multifactorial and includes familial or genetic factors, viruses, drugs, and adaptive changes with normal or reduced renal mass.^[3]^ Pembrolizumab is an antiprogrammed death 1 (PD-1) immunoglobulin G4 antibody approved for the treatment of advanced melanoma and nonsmall cell lung cancer. As an immune checkpoint inhibitor (CPI), pembrolizumab can cause various immune-related adverse events (AEs), including nephritis and interstitial nephritis.^[4]^ Several cases of anti-PD-1 therapy-induced glomerulonephritis have been reported thus far, but FSGS has seldom been reported. This report describes the case of a 46-year-old woman diagnosed with FSGS who had previously undergone treatment for malignant melanoma with pembrolizumab.

## 2 Case presentation

A 46-year-old Korean woman presented with a 1-month history of progressive generalized edema since November, 2020. She had a history of malignant melanoma on the left posterior side of the thigh that was treated with wide local excision on May 29, 2019. Because of metastasis to the left sentinel and inguinal lymph nodes, she received pembrolizumab 200 mg every 21 days for 1 year, from July 10, 2019 to July 3, 2020. During pemblizumab treatment, she was started on levothyroxine for hypothyroidism, presumably an immune-related AE of pembrolizumab. Four months after the cessation of pembrolizumab, she developed generalized edema, most notable in the periorbital area and both hands and legs, which gradually worsened. She denied taking any new medication or personal and family history of kidney disease. Her baseline observations on admission were as follows: height, 165 cm; weight, 70 kg; blood pressure, 140/80 mm Hg; heart rate, 74 bpm and regular; and temperature, 36.4°C. Clinical examination revealed grade 3 pitting edema in both lower legs. Four months before the onset of the edema, her blood pressure was 120/70 mm Hg, body weight was 65 kg, and body mass index was 23.88 kg/m^2^. Laboratory findings were as follows: white blood cell count 10,940/μL (reference range 3800–11,000/μL); hemoglobin 12.5 g/dL (reference range 11.2–15.0 g/dL); platelet count 208 × 10^3^/μL (reference range 140–420 × 10^3^/μL); total protein 5.71 g/dL (reference range 6.0–8.0 g/dL); albumin 2.84 g/dL (reference range 3.3–5.2 g/dL); blood urea nitrogen 5.4 mg/dL (reference range 6–26 mg/dL); creatinine 0.66 mg/dL (reference range 0.4–1.2 mg/dL); estimated glomerular filtration rate 105.9 mL/min per 1.73 m^2^; total cholesterol 238 mg/dL (reference range 0–200 mg/dL); urine red blood cells 6 to 10 cells/high power field (reference range 0–2 cells/high power field); and urine protein to creatinine ratio 3277 mg/g (reference range 0–150 mg/g). Urine dipstick for protein was negative before pembrolizumab treatment. Human immunodeficiency virus antigen and antibody results were negative. A contrast-enhanced computed tomography scan of the abdomen showed normal sized kidneys (right kidney: 9.6 cm; left kidney: 10.7 cm) and no evidence of vaso-occlusive processes in the renal arteries.

**Figure 1:**
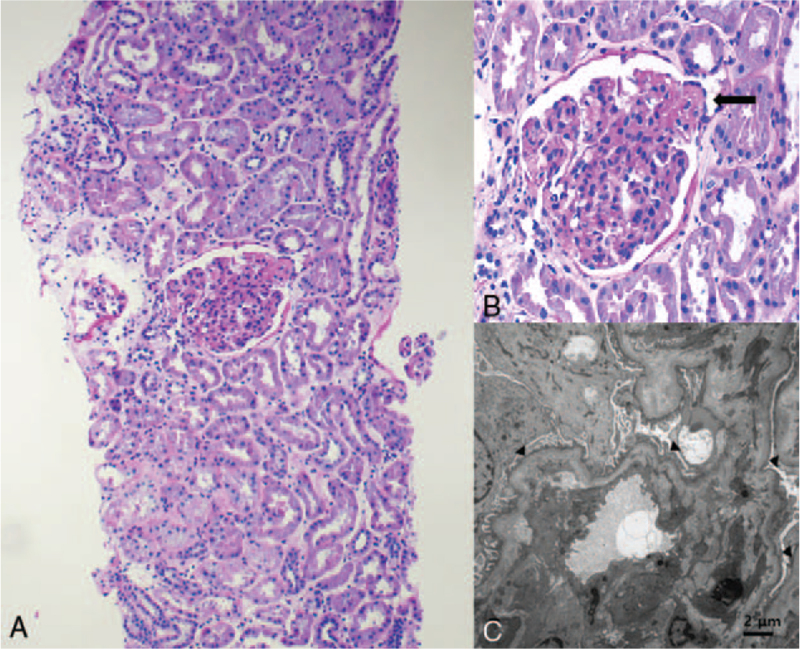
Kidney histopathology. (A) Light microscopic image of the kidney biopsy specimen showing focal glomerulosclerosis with atrophied tubules and fibrosis in the interstitium (10× magnification). (B) Light microscopic image of the kidney biopsy specimen showing focal glomerulosclerosis (arrow). (C) Electron microscopic image of the kidney showing widely effaced epithelial cell foot processes (arrow) (400× magnification).

She underwent kidney biopsy for suspected glomerulonephritis. Light microscopic findings of biopsy specimens showed that up to 36 glomeruli, 2 glomeruli exhibited segmental sclerosis with atrophied tubules and fibrosis in the interstitium (Fig. 1A, B). Electron microscopy revealed wide effacement of the epithelial cell foot processes (Fig. 1C). Immunofluorescence microscopy showed no immune complexes or autoantibody deposition.

After ruling out other causes of secondary FSGS, she was diagnosed with FSGS caused by pembrolizumab. The patient was started on irbesartan and furosemide to treat high blood pressure, proteinuria, and edema. She did not resume treatment with pembrolizumab and immunosuppressive therapy was not applied. After 2 months, the features of nephrotic syndrome resolved and the urine protein-to-creatinine ratio decreased to 203 mg/g.

## 3 Discussion and conclusions

FSGS presents a specific histologic pattern of glomerular injury and can be broadly divided into primary (idiopathic) or secondary forms.^[1]^ Although the causes of FSGS are multifactorial, the most common form is primary FSGS caused by circulating permeability factors and accounts for 80% of cases.^[2]^ Other causes include genetic mutations in podocyte components, viral infections, drug toxicities, and adaptation of structural-functional responses. Regardless of etiology, the central pathogenesis is directed or inherent podocyte injury.^[5]^

Among the CPIs, pembrolizumab is an anti-PD-1 antibody that acts by disrupting the engagement of PD-1 with its ligands and impeding inhibitory signals that lead to the recognition of tumor cells by cytotoxic T cells. However, CPI treatment may also impair the self-tolerance of the immune system and has been associated with an increased risk of developing immune-related AEs. Immune-related AEs can affect multiple organs of the body and are most commonly seen in the skin, gut, liver, lung, and thyroid, as well as the endocrine, musculoskeletal, nervous, hematologic, cardiovascular, and renal systems. The estimated incidence of renal immune-related AEs was 2% to 4.5% in patients treated with CPIs.^[4]^ The vast majority of renal AEs are acute kidney injury. Acute tubulointerstitial nephritis is the most commonly reported CPI-related renal pathology. However, lupus nephritis, thrombotic microangiopathy, membranous nephropathy, pauci-immune glomerulonephritis, FSGS, minimal change disease (MCD), and IgA nephropathy have also been reported in the literature.^[6–11]^ The mechanism behind podocyte injury by PD-1 inhibitors has not been established, but suggested mechanisms include the development of autoimmunity to kidney self-antigens by modulating humoral immunity in addition to T-cell-mediated immunity and release of cytokines leading to podocyte foot process effacement.^[12–14]^

To date, there have been 5 reported cases of CPI-induced MCD and FSGS (Table 1). Three of the cases reported MCD induced by pembrolizumab or ipilimum.^[15,16]^ The grades of renal toxicities ranged from 1 to 3 and the CPIs were stopped, and steroid therapies were applied in all cases, resulting in complete or partial recovery of the kidneys. Two of the cases reported FSGS induced by nivolumab.^[4,17]^ The grades of renal toxicities were both 2. Nivolumab was discontinued, and steroids with or without mycophenolate mofetil were administered, resulting in partial recovery.

**Table 1:** Reported cases of immune checkpoint inhibitor-induced minimal change disease and focal segmental glomerulosclerosis.

Case	Renal pathology	Malignancy	CPI duration	Grade of renal toxicities	Management	Renal Outcome
Angelika et al (2016)^[15]^	MCD	Malignant pleural mesothelioma	Pembrolizumab 6 weeks	G2	CPI discontinued’ and ’Prednisone ∼ (1 mg/kg)	Complete recovery
Kitchlu et al (2017)^[16]^	MCD	Hodgkin lymphoma	Pembrolizumab 4 weeks	G3	CPI discontinued’ and ’Prednisone ∼ (1 mg/kg)	Partial recovery
Kitchlu et al (2017)^[16]^	MCD	Melanoma	Ipilimumab 18 months	G1	CPI discontinued’ and ’Prednisone ∼ (1 mg/kg)	Complete recovery
Daanen et al (2017)^[17]^	FSGS	RCC	Nivolumab 2 months	G2	CPI discontinued’ and ’Prednisone ∼ (1 mg/kg) MMF 750 mg twice daily	Partial recovery
Mamlouk et al (2019)^[4]^	FSGS’ and ’ATIN with eosinophils	RCC CML	Nivolumab 14 months	G2	CPI discontinued’ and ’Prednisone ∼ (0.8 mg/kg)	Partial recovery

The histology of MCD shows no glomerular lesions or only mild focal mesangial prominence not exceeding 3 or 4 cells per segment.^[18]^ In our case, kidney biopsy showed segmental glomerular sclerosis with atrophied tubules and interstitial fibrosis, we excluded MCD.

To our knowledge, this is the first report of a patient presenting with FSGS after treatment with pembrolizumab. According to the management of immune-related AEs in patients treated with CPI therapy (American Society of Clinical Oncology Clinical Practice guideline),^[19]^ which is based on grading the severity of renal toxicities only by serum creatinine levels and not including proteinuria because glomerular disease caused by CPIs are rare, the grade of renal toxicity was 1 and milder than other reported cases of FSGS. Because of the paucity of available data, recommendations on management of proteinuria and glomerular diseases in patients on CPIs does not yet exist. Vanoverbeke et al^[20]^ recommended that kidney biopsy is mandatory in patients with heavy proteinuria (>3.5 g/day) and may recommend in proteinuria between 1 and 3.5 g/day, and withhold therapy if a glomerular disease related to immunotherapy is diagnosed on kidney biopsy. She did not resume the treatment of pembrolizumab and was treated with irbesartan and furosemide. The kidneys recovered completely without immunosuppressive therapies.

According to the Kidney Disease Improving Global Outcomes Clinical Practice Guideline for Glomerulonephritis,^[21]^ complete recovery or complete remission of MCD and FSGS refers to reduction of proteinuria to <0.3 g/day (<300 mg/g). Partial recovery or partial remission of the diseases refers to reduction of proteinuria to 0.3 to 3.5 g/day (300–3500 mg/g) or a decrease in proteinuria by at least 50% from baseline and <3.5 g/day. Proteinuria was the predictor of renal recovery following CPI therapy in reported cases.

Mamlouk et al^[4]^ reported 16 patients with renal immune-related AEs from 2008 to 2018 who were treated with CPIs and subsequently underwent a kidney biopsy at a single center. There were various types of nonrenal immune-related AEs, with the most common being hypothyroidism. After or at the time of nonrenal immune-related AE diagnosis, nephrotoxicity was observed in most cases. Consistent with previous reports, the patient in our case developed hypothyroidism as a nonrenal immune-related AE prior to renal immune-related AE.

In conclusion, FSGS can occur as a renal immune-related AE in patients treated with PD-1 inhibitors. Therefore, patients treated with PD-1 inhibitors should undergo evaluation of renal function and urinalysis at baseline and after treatment. A renal biopsy would be recommended in patients presenting with renal injury and/or unexplained proteinuria >1 g/day to determine the underlying cause and establish an appropriate therapeutic plan.

## Author contributions

**Conceptualization:** Eun Young Seong.

**Data curation:** Sungmi Kim, Wanhee Lee.

**Investigation:** Da Woon Kim, Hakeong Jeon.

**Project administration:** Hyo Jin Kim, Harin Rhee.

**Supervision:** Eun Young Seong, Sang Heon Song.

**Validation:** Eun Young Seong, Sang Heon Song.

**Writing – original draft:** Da Woon Kim, Hakeong Jeon.

**Writing – review & editing:** Eun Young Seong.
